# New Layered Boride NiPtB_2–*x*_ (*x* = 0.5) with a Ternary Derivative Structure of MoB

**DOI:** 10.1021/acs.inorgchem.4c04399

**Published:** 2025-01-27

**Authors:** Leonid Salamakha, Oksana Sologub, Berthold Stöger, Herwig Michor, Neven Barisic, Peter F. Rogl, Ernst Bauer

**Affiliations:** † Institute of Solid State Physics, TU Wien, A-1040 Vienna, Austria; ‡ X-Ray Centre, TU Wien, A-1060 Vienna, Austria; § Institute of Materials Chemistry, University of Vienna, A-1090 Vienna, Austria; ∥ Department of Physics of Metals, Faculty of Physics, I. Franko L’viv National University, 79005 L’viv, Ukraine; ⊥ Department of Physics, Faculty of Science, University of Zagreb, HR-10000 Zagreb, Croatia

## Abstract

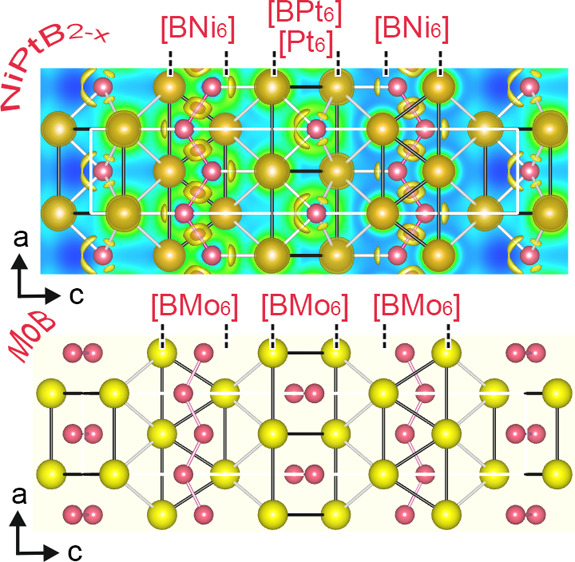

A novel ternary boride, NiPtB_2–*x*_ (*x* = 0.5), was obtained by argon-arc melting of the elements followed by annealing at 750 °C. It exhibits a new structure type with the space group *Imma* (*a* = 2.9835(3) Å, *b* = 3.0470(3) Å, *c* = 15.3843(3) Å; *Z* = 4; single-crystal X-ray data) and displays distinct layers of condensed [BNi_6_] and [BPt_6_] (and [Pt_6_]) trigonal prisms with mutually perpendicular axes. Atoms of Pt and Ni from adjacent layers interlink to form empty tetragonal pyramids and tetrahedra. Two boron atom positions form two orthogonal zigzag chains; however, one boron position exhibits a partial boron occupancy. Considering B-deficiency, the platinum boride substructure in NiPtB_2–*x*_ quantitatively corresponds to a trigonal prismatic slab in the Pt_2_B structure, while the nickel boride partial structure is consistent with the CrB-type NiB binary. Cell parameters and atomic coordinates of NiPtB_2–*x*_ and Pt_2_B were refined in the scope of generalized gradient approximation. Chemical bonding analysis by means of the electron localizability approach, supported by Bader charge analysis, reveals a strong electron contribution of Ni atoms for stabilization of the boron zigzag chains, wherein boron atoms are bonded covalently. Bonding within the platinum boride partial structures in the studied compounds varies depending on the atom coordination of boron: from covalent in both the NiPtB_2–*x*_ structure and trigonal prismatic slabs in Pt_2_B to mixed metallic with covalent contributions in [BPt_6_] octahedra in Pt_2_B. Electrical resistivity measurements characterize NiPtB_2–*x*_ as a metal with no phase transitions in the temperature range from 2 to 300 K, in concord with electronic band structure calculations and specific heat measurements. The compound is characterized by a positive Hall coefficient at 20 K. This work unveils a new elemental space on realizing novel layered boride structural arrangements and provides a reference for future experiments.

## Abstract 2

The family of layered borides was extended to NiPtB_2−*x*_ (space group *Imma*), a new ternary derivative structure of MoB (space group *I4_1_/amd*).

## 1 Introduction

In the past decade, a large number of research groups have actively engaged in the study of layered compounds containing boron as one component and having transition metals and *p*-elements as the second and third components, respectively. Notable examples are MAlB and M_2_AlB_2_ borides^1−3^ (M = transition metal, MoAlB-type, space group *Cmcm*,^4^ and Fe_2_AlB_2_ (Mn_2_AlB_2_)-type, space group *Cmmm*,^5,6^ respectively), due to their structural similarities to the MAX phases (M_*n*+1_AX_*n*_),^7−10^ where the M_*n*+1_X_*n*_ sublattice interleaves with monolayers of the A element (M = early transition metal, A = 12 to 16 group element, and X = C, N, B and/or P). These structural arrangements, along with the included elements, often result in attractive metallic and ceramic properties that combine those of MX binary counterparts and the A component and allow for the enhancement/modification of these characteristics by various routes of materials design.^3,11,12^

The boride substructure of CrB is known as a building fragment of MAB phases, where the A layers are located between the layers of the M/B substructure.^3,4,13−15^ According to the different numbers of metal layers in the structures, different compositions are obtained, e.g., the compounds of MoAlB-, Mn_2_AlB_2_-, Cr_3_AlB_4_-, Cr_4_AlB_6_-, Cr_4_AlB_4_-, and Ru_3_Al_2_B_2_-types. All these structures are orthorhombic and exhibit a parallel arrangement of the boron subunits. The complexity of the boride substructure is limited to motifs of CrB-type (−B–B– chains) and AlB_2_-type (chains of B_6_ hexagons). Even though CrB-type compounds are well-represented in both binary and ternary boride systems, the alloying metals of reported MAB phases are confined to elements, found in traditional ternary MAB phases. Lately, however a number of chemically and structurally different phases such as hexagonal Ti_2_InB_2_,^16^ tetragonal Y_5_Si_2_B_8,_^17^ hexagonal Cr_5_Si_3_B,^18^ hexagonal i-MAB phases,^19^ and monoclinic Ni_n+1_ZnB_n_^15,20,21^ have been classified as MAB phases due to their layered structural arrangements and common with MAX phases' physical properties (high electrical conductivity,^22^ stiffness,^22^ and resistance to thermal shock).^18^

In contrast to CrB-type, MoB (space group *I*4_1_/*amd*, *a* = 3.105 Å, *c* = 16.97 Å, Mo in 8*e* (0,,*z*; *z* = 0.179), B in 8*e* (0,,*z*; *z* = 0.023)) is a much less common type of boride.^23^ The compounds of this structure type exist in M-B systems (M = Cr, Mo, W) as low-temperature phases in rather small homogeneity ranges.^24−26^ Early reports on ternary boride systems involving MoB type were mainly concerned with the solubility of M elements (M = Cr, Nb, V) in WB and MoB.^27^ The existence of MoB-type ternary phases showing a statistical occupation of the Mo atom position by Re/Cr and Re/V in Re-{Cr,V}-B systems was indicated by Kuzma.^27^ The structure of MoB is more complex than that of CrB and exhibits four trigonal prismatic [MoB_6_] layers per unit cell, which alternate along the *c*-axis; the −B–B– chains within adjacent Mo–B blocks are orthogonal. In contrast to CrB-type-originated MAB phases, the number of ternary MoB-type-based phases is small, among them, e.g., Ru_2_ZnB_2–*x*_^13^ is the only MAB phase containing an orthogonal arrangement of −B–B– chains.^10^ It is thus interesting to explore whether there is any new elemental combination that may produce structures exhibiting the stacking sequence of fragments similar to the MoB-type MAB phase.

With this intention, a new compound in the Ni–Pt–B system, NiPtB_2–*x*_ (*x* = 0.5), was synthesized and studied, employing single-crystal and powder X-ray diffraction, first-principles calculations, electrical resistivity, Hall coefficient, and specific heat measurements. The results obtained open new perspectives on realizing MAB phases with new structural arrangements and elemental compositions and allow for further potential of tuning properties.

## 2 Experimental Section

### 2.1 Synthesis

Several alloys of 0.8 g each within the concentration range Ni_20_Pt_20_B_60_ (at %)–Ni_30_Pt_30_B_40_ (at %) were synthesized from high-purity elements (Ni pieces 99.999 mass% or Ni foil 99.99 mass % and crystalline boron 99.8 mass %, all obtained from ChemPur, Germany, and Pt foil 99.99 mass %, obtained from Ögussa, Austria) by arc melting on a water-cooled copper hearth under a Ti-gettered argon atmosphere. The charges were fused together under a weak arc and melted; afterward, the alloys were flipped three times and remelted to ensure complete fusion. The total mass losses after melting were below 0.5 mass%, and no corrections were necessary. The arc-melted buttons were cut into pieces, where one piece was wrapped in tantalum foil and vacuum-sealed in a quartz tube for annealing at 750 °C for 240 h.

### 2.2 Techniques Used for Characterization

Powder X-ray diffraction (XRD) data were collected employing a Guinier-Huber image plate system with monochromatic Cu K_α1_ radiation (8° ≤ 2θ ≤ 100°) from as-cast and annealed alloys. Quantitative Rietveld refinements of the powder XRD data were performed with the FULLPROF program^28^ with the use of its internal tables for atom scattering factors. The annealed samples were polished applying standard procedures and examined by scanning electron microscopy (SEM) using a Philips XL30 ESEM with an EDAX XL-30 EDX-detector. As accurate quantitative boron determination is not possible with EDX detection, the measurements were focused on determining the Ni:Pt ratios. The X-ray powder diffraction data, together with SEM-EDX data analysis, unambiguously indicated the existence of an unknown ternary nickel platinum boride phase with a 50Ni:50Pt ratio in all three alloys studied (Ni_20_Pt_20_B_60_ (at %), Ni_25_Pt_25_B_50_ (at %), and Ni_30_Pt_30_B_40_ (at %)). For single-crystal X-ray diffraction studies, a four-circle STOE Stadivari diffractometer equipped with an Eiger CdTe hybrid photon counting detector (Euler geometry, Mo Kα radiation) was used. Orientation matrices and unit cell parameters were derived with the help of the diffractometers’ software; the data were scaled using the multiscan approach implemented in LANA.^29^ Space group determination, structure solution, and refinement were performed employing the WinGX program package.^30−33^ Further details concerning the single-crystal X-ray diffraction experiments are summarized in Table 1. A detailed description of the structural refinement is given below. The arc-melted and annealed ingot of NiPtB_2–*x*_ was polished to obtain a bar-shaped sample (about 1 × 1 × 5 mm^3^) on which temperature-dependent electrical resistivity was studied using a four-probe dc method in the range from room temperature down to 2 K and in magnetic fields up to 8 T with the Quantum Design PPMS. Field-dependent Hall resistivity was studied using the device mentioned above. The magnetic field for the Hall effect was applied perpendicularly to the electric current. The measured transverse voltage in the Hall experiment was collected with the sample orientation reversed and antisymmetrized to remove the spurious remainder of the longitudinal signal. Specific heat measurements were performed in the temperature range 2–300 K employing a thermal-relaxation technique.

**Table 1:** Structure Refinement Details from Single-Crystal XRD

formula from refinement	NiPtB_1.48(9)_
theta range (deg)	2.65 < θ < 36.73
crystal size (μm)	45 × 48 × 60
space group	*Imma* (No. 74)
*a* (Å)	2.9835(3)
*b* (Å)	3.0470(3)
*c* (Å)	15.3843(3)
*Z*	4
mosaicity	<0.50
reflections collected/unique	3379/227
number of variables	16
reliability factors	*R*_1_ = 0.0358
	*wR*_2_ = 0.0804
GOF	1.086
residual density; max; min (e^–^/Å^3^)	4.087; −6.378

### 2.3 Electronic Structure Calculations and Chemical Bonding Analysis

Band structure (electron dispersion) and density of states (eDOS) calculations were performed within the DFT framework using the Quantum ESPRESSO 6.7 package^34^ on the modeled structure described below in Section 3.2. Correlation and exchange effects of the electrons were handled utilizing the generalized gradient approximation (GGA) of Perdew, Burke, and Ernzerhof, revised for solids (PBEsol).^35^ Electron-ion interactions were treated using both fully relativistic and pseudorelativistic projector augmented wave (PAW)^36,37^ potentials constructed according to the code supplied by the PSLibrary (version 1.0.0).^38^ For nickel and platinum, 3*s*- and 3*p*-, and 4*s*-, 4*p*-, and 4*f*-electrons, respectively, were considered as valence states. The electron wave functions were expanded into plane waves with a kinetic energy cutoff of 100 Ry. For the charge density, a kinetic energy cutoff of 800 Ry was used. The unit cell parameters and atomic positions of NiPtB_2–*x*_ were relaxed using the Broyden–Fletcher–Goldfarb–Shanno (BFGS) algorithm and a 15 × 15 × 3 k-point mesh constructed using the Monkhorst–Pack method^39^ that guarantees less than 0.15 Å^–1^ spacing between the k-points. The convergence threshold for self-consistent-field iteration was set at 10^–9^ eV. A denser grid of 30 × 30 × 6 was used for the calculation of the electronic density of states (DOS) and the electron localization function (ELF). Calculations of Pt_2_B^40^ were performed on a 3 × 15 × 12 k-point grid according to the procedure outlined above. The ELF distribution was analyzed and visualized using the VESTA v.3.5.8 software.^41^

### 2.4 Crystal Structure Determination from Single-Crystal XRD Data

XRD data for the crystal, which was selected from the alloy Pt_25_Ni_25_B_50_ annealed at 750 °C, were indexed with a body-centered orthorhombic unit cell with lattice parameters *a* = 2.9835(3) Å, *b* = 3.0470(3) Å, and *c* = 15.3843(3) Å. Systematic absences were consistent with two space groups, *Imma* and *Ima*2. Beginning with the centrosymmetric space group *Imma*, direct methods^32^ were used to determine the positions of the heavier Pt and Ni atoms. Further differential Fourier calculations^33^ allowed for the localization of boron atoms. The site occupation factors for Pt and Ni atoms, refined without constraints, indicated full occupancy, resulting in the formula NiPtB_2_ with four formula units per unit cell. Subsequent refinement steps were performed by determining anisotropic ADPs for Pt and Ni, but isotropic for boron atoms, yielding an enlarged value of the displacement parameter for B2 compared to B1. This suggests a possible fractional population of the B2 atom position. The variable occupancy of the B2 site was refined in further steps of refinements, leading to a reasonable ADP value at a population level of ∼48%, thus delivering the formula NiPtB_1.48(9)_. The compound’s formula will be further written in its simplified form as NiPtB_2–*x*_, where *x* has a value of 0.5. The refinement converged to a reliability factor value of 0.0358 exhibiting a residual electron density of 4.087 e/Å^3^ at 0.54 Å (in 8*i* (*x*,,*z*) *x* = 0.1768, *z* = 0.0834) to the Pt atom (Table 1). The attempt to introduce this electron density as a split site for Pt1 resulted in occupation parameters of 0.98/0.02 for Pt1/Pt11 and slightly reduced the residual electron density (3.49 e/Å^3^ at 0.42 Å from Pt11) but did not affect the reliability factor values and revealed short distances between crystallographically equivalent Pt11 atoms (0.7316 Å). At this point, the collected data were also processed in the space group *Ima*2 (no. 46). The refinement resulted in *R*_F_ = 0.0357 and showed a peak of 5.16 e/Å^3^ at 0.50 Å in 4*b* (,*y*,*z*) *y* = 0.5819, *z* = 0.1640). Refinement of Pt in split Pt1/Pt11 prompted a more chemically sound solution for the local disorder (0.93Pt1/0.07Pt11; *d*_Pt1-Pt11_ = 2.64(2) Å, *d*_Pt11-Pt11_ = 2.78(1) Å *R*_1_ = 0.0353) but did not eliminate the problem of residual electron density, which remained at 4.42 e/Å^3^ at 0.43 Å from Pt11. The small differences between the solutions in the centrosymmetric group and noncentrosymmetric *Ima*2 also concerned the atom positions of B1 and B2, which both showed a slight drift along the *c*-axis due to the free *z* parameter of the 4*b* (*,y,z*) site in the space group *Ima*2 (i.e., *z* = 0.01(1) for B1 and *z* = 0.02(2) for B2). Except for these small deviations, refinement in *Ima*2 revealed Pt and Ni atoms in the Wyckoff positions corresponding to those in the space group *Imma*. Moreover, the test for higher symmetry applying PLATON^30^ indicated the space group *Imma*. As the obtained atomic coordinates coincide in both centrosymmetric and noncentrosymmetric space groups, and no reduction of residual electron density upon refinement of split atom sites and no improvement of reliability factors have been observed, we retain the structure description within the ordered structure model without deviation from centrosymmetry. The residual electron densities obviously reflect Fourier ripples to the large Pt-peak, which have been frequently observed in crystals containing 4*d*- or 5*d*-metals, e.g., Pt_2_B,^40^ La_3_Pd_25_B_8_,^42^ La_3_Ru_8_B_6_,^43^ and EuPt_4_B.^44^

**Figure 1:**
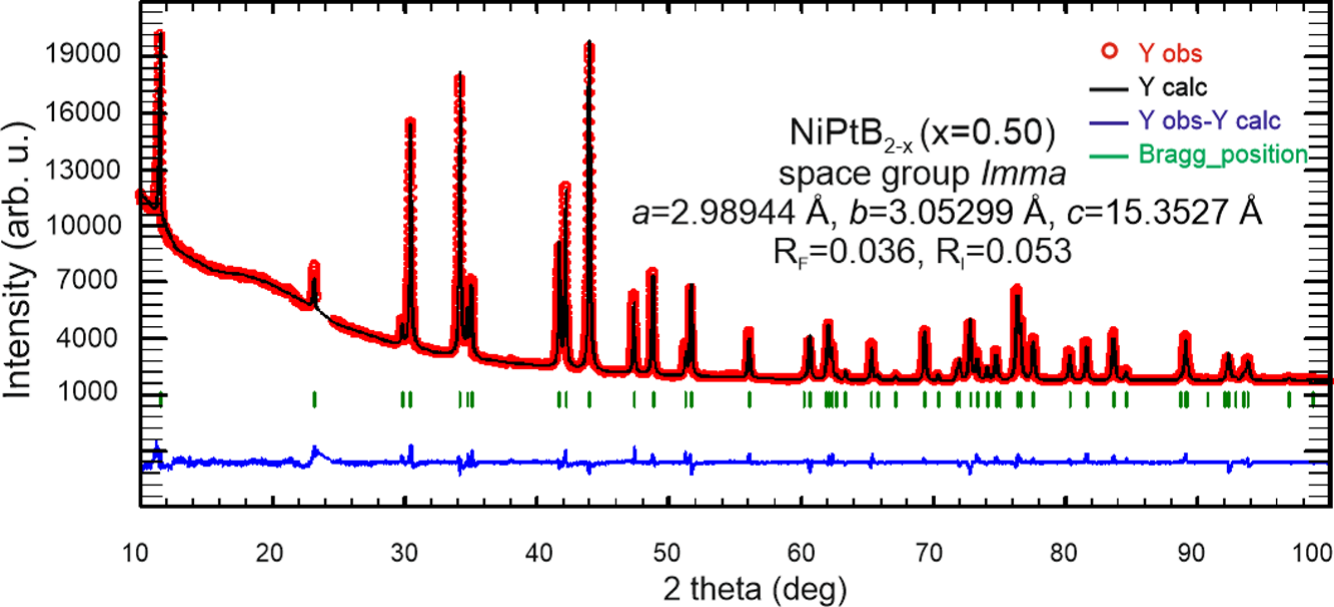
Rietveld refinement of powder X-ray diffraction intensity data of NiPtB_2–*x*_ annealed at 750 °C. The excluded region contains a small peak from the sample holder.

X-ray powder diffraction intensities collected from the polycrystalline alloys with a nominal composition of NiPtB_2–*x*_ (*x* = 0.5) are in best agreement with the intensities calculated from the structural model taken from single-crystal data, as inferred from Rietveld refinement (Figure 1). The final positional and displacement atom parameters and interatomic distances obtained from single-crystal data are listed in Tables 2 and 3.

**Table 2:** Atomic Coordinates^a^ and Displacement Parameters

Pt1	4*e* (0,,*z) z* = 0.07549(2)
Occ.	1.00
U_11_, U_22_, U_33_^b^	0.0139(3), 0.0115(3), 0.0132(3)
Ni1	4*e* (0,,*z) z* = 0.6839(1)
Occ.	1.00
U_11_, U_22_, U_33_	0.0140(6), 0.0113(5), 0.0139(5)
B1	4*e* (0,,*z) z* = 0.2176(8)
Occ.	1.00
U_11_, U_22_, U_33_	0.015(4), 0.012(4), 0.015(5)
B2	4*e* (0,,*z) z* = 0.5293(13)
Occ.	0.48(9)
U_iso_^b^	0.010(3)

a Crystal structure data are standardized using the program Structure Tidy.^45^

b Anisotropic (U_*ij*_) and isotropic (U_iso_) atomic displacement parameters are given in Å^2^; U_23_ = U_13_ = U_12_ = 0.

**Table 3:** Selected Interatomic Distances (in Å)

Pt1–	B1	2.19(1)	Ni1–	2B1	2.149(9)
	2B2^a^	2.20(1)		4B1	2.194(3)
	4B2	2.248(7)		B2	2.38(2)
	4Ni1	2.7069(10)		2Ni1	2.523(3)
	2Pt1	2.7779(6)		4Pt1	2.7069(10)
B2–	2B2	1.77(2)	B1–	2B1	1.79(1)
	2Pt1	2.20(1)		2Ni1	2.149(9)
	4Pt1	2.248(7)		Pt1	2.19(1)
	Ni1	2.38(2)		4Ni1	2.194(3)

a The distances involving B2 may not occur at ∼50% occupancy B2.

## 3 Results and Discussion

### 3.1 Structural Description and Analysis of NiPtB_2–*x*_

**Figure 2:**
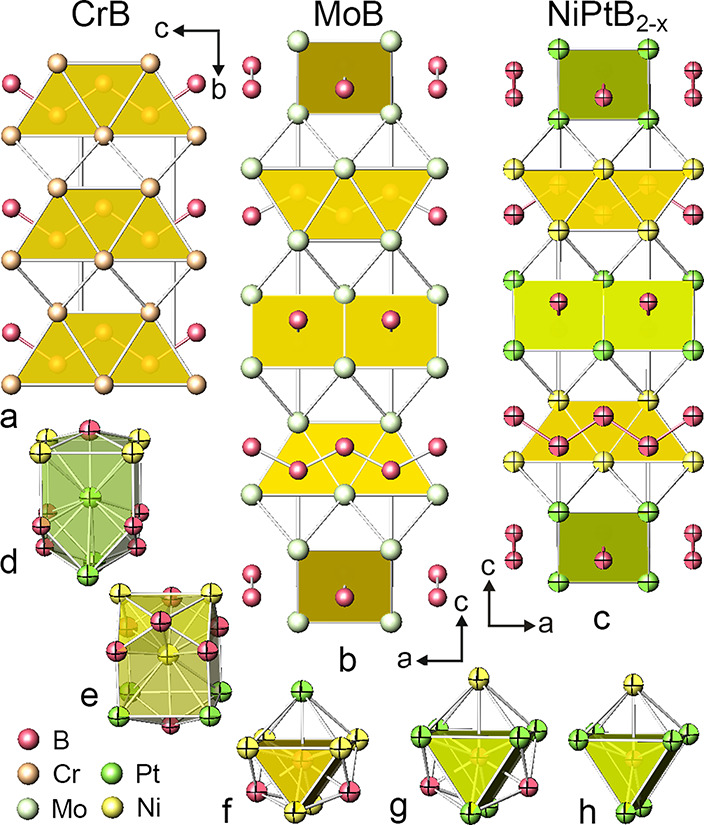
CrB (a), MoB (b), and NiPtB_2–*x*_ (c) structures as arrangements of layers of condensed [BM_6_] trigonal prisms. Coordination polyhedra of Pt (d), Ni (e), B1 (f), and B2 (g). The figures reflect the presence of boron in each Pt_6_ trigonal prism of NiPtB_2–*x*_. The coordination polyhedron of B2 assumes the presence of boron in every second platinum trigonal prism (h).

The crystal structure of the new ternary structure type of borides NiPtB_2–*x*_ is shown in Figure 2 in comparison with the CrB and MoB structures. The atoms in NiPtB_2–*x*_ are represented by their thermal ellipsoids. There are two crystallographically independent metal atom sites (one platinum and one nickel) and two boron atom sites, of which the B2 atom site is prone to defects. Trigonal prisms M_6_ share triangular and rectangular faces to form infinite layers arranged perpendicularly to the *c*-axis. Each layer is built by one kind of metal atom, either Pt or Ni. Prism axes in adjacent layers are perpendicular; the atoms of Pt and Ni from neighboring layers interlink to form empty tetragonal pyramids and tetrahedra. The coordination polyhedra around the boron atoms in the nickel boride substructure (B1) are three-capped trigonal prisms [B1B_2_Ni_6_Pt_1_] (Figure 2f); the nearest neighbor boron atoms form one-dimensional zigzag chains. The coordination polyhedron around the nickel atoms exhibits 13 vertices (Figure 2e), among which seven vertices are decorated by boron atoms, including the one filled with B2, which exhibits a 50% level of occupation. In the case of full occupation of the B2 atom site, the coordination numbers and the shape of coordination polyhedra of atoms in the platinum boride substructure replicate those of the nickel boride layers, demonstrating, however, the differences in interatomic distances (Figure 2d,g and Table 3). The 50% occupation of the B2 atom position allows the assumptions that in the trigonal prismatic platinum boride layers, every second Pt trigonal prism is filled with boron (Figure 2h) as well as that, due to the statistical occupation of the B2 atom position, the situation may occasionally occur when a portion of adjacent Pt trigonal prisms is filled with boron.

The NiPtB_2–*x*_ structure (Figure 2c) is a ternary-ordered derivative structure of the binary MoB type (space group *I*4_1_/*amd*; Mo in 8*e* (0,,*z*), B in 8*e* (0,,*z*)). In the MoB type, B atoms are located in Mo_6_ trigonal prisms, which condense to form layers via triangular and rectangular faces (Figure 2b). The same layers of boron-filled M_6_ trigonal prisms have been observed in the CrB-type structures (Figure 2a); however, in MoB, the prisms are related via symmetry operations of the *I*4_1_/*amd* space group, resulting in layers of trigonal prisms with mutually perpendicular axes alternating along the *c-*axis. Boron atoms in both structures interlink to form zigzag chains; while in the CrB structure all the −B–B– chains are parallel, in MoB the boron chains in adjacent layers are orthogonal.

**Figure 3:**
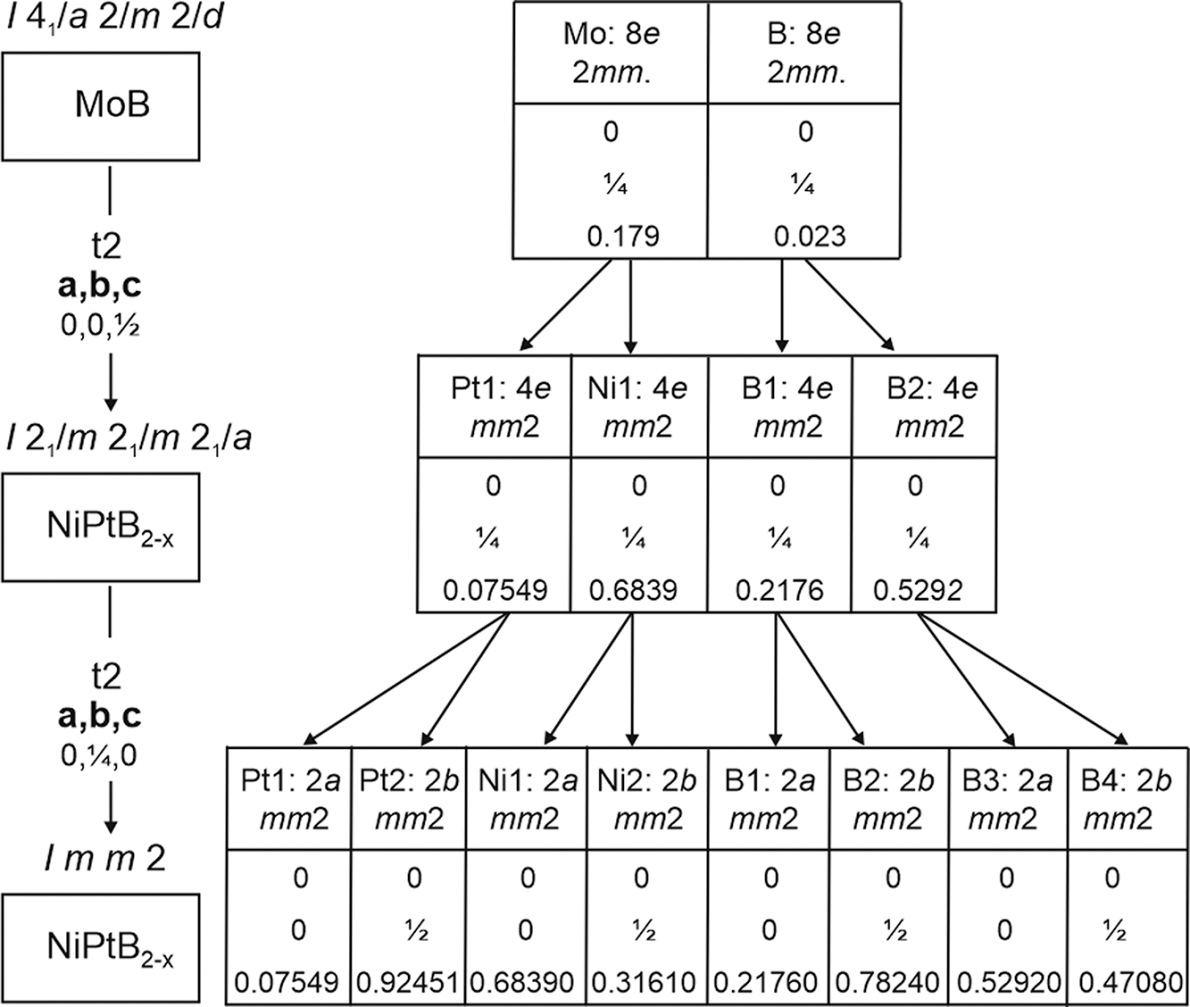
Bärnighausen^48^ scheme of the group–subgroup relationships for the structures of MoB (space group *I*4_1_/*amd*), NiPtB_2–*x*_ (space group *Imma*) (determined experimentally), and NiPtB_2–*x*_ (space group *Imm*2) (used in DFT calculations).

The NiPtB_2–*x*_ structure crystallizes in the space group *Imma*, which is a *translationengleiche* subgroup of index 2 of space group *I*4_1_/*amd* (Figure 3). The reduction of symmetry leads to a splitting of the Mo (8*e*) and B (8*e*) atom positions of MoB in two four-fold atom sites each, of which two metal atom sites are occupied in an ordered manner by Pt and Ni, rendering the formation of two distinct layers of condensed trigonal prisms (Ni_6_ and Pt_6_) in the structure of NiPtB_2–*x*_. The boron atom position within the layer composed of platinum trigonal prisms is half-vacant; assuming full occupation of the B2 atom site, the B2–B2 distance in the zigzag chain is 1.77(2) Å. The B1–B1 distance in the zigzag chain within [BNi_6_] blocks is 1.79(1) Å. These distances are comparable to other well-know chain borides, i.e., 1.738 Å in MoB,^23^ 1.780 Å in CrB,^46^ 1.83 Å in NbCoB_2_,^47^ 1.834 Å in MoAlB,^4^ and 1.762 Å in Cr_2_AlB_2,_^10^ etc.

Apart from the binary MoB structure, the orthogonal arrangement of -B-B- chains exists in Ru_2_ZnB_2–*x*_,^13^ while the arrangement of planar infinite layers of [BM_6_] trigonal prisms with perpendicular axes in adjoining subunits occurs, for example, in CuIr_2_B_2–*x*_^49,50^ and Ir_4_ZnB_3_ (Figure S1).^51^ The Ru_2_ZnB_2–*x*_ structure (*x* = 0.6, space group *I*4_1_/*amd*^13^) was found to form by alternatively stacking of layers of Ru trigonal prisms partially filled with B and Zn layers; the trigonal prisms are mutually rotated by 90° in subsequent layers. In CuIr_2_B_2–*x*_ (*x* = 0.52, space group *Cmcm*),^49,50^ the iridium atoms form layers of trigonal prisms which condense via rectangular faces in a block; the prism axes in subsequent layers are perpendicular. The blocks of three trigonal prismatic layers are interleaved with two planar layers of copper. Two of the three layers in the boride substructure exhibit an ordered type of boron defects with no boron–boron contact, while the remaining one reveals disordered vacancies of boron atoms, assuming the possible formation of -B-B- chain fragments. In the Ir_4_ZnB_3_ structure (space group *Pmmm*), the blocks of three iridium boride trigonal prismatic layers alternate with one Zn layer. In this structure, due to boron vacancy ordering in the boride substructure, no direct contacts for boron atoms occur. By the disordered distribution of B vacancies in the platinum boride layer, the NiPtB_2–*x*_ structure is related to Ru_2_ZnB_2-x_ and CuIr_2_B_2–*x*_. The new structure NiPtB_2–*x*_ is located on the line, extending from binary NiB (CrB-type structure) to the recently discovered Pt_2_B (space group *C*2/*m*)^40^ in the Ni–Pt–B phase diagram and shares many similarities with the structure of the platinum boride binary compound as well. A thorough description of the Pt_2_B crystal structure can be found in our prior work,^40^ and its comparison with NiPtB_2–*x*_ is addressed in Section 3.3.

### 3.2 Density Functional Theory Calculations

To perform DFT calculations on NiPtB_2–*x*_, a model with an ordered distribution of boron atoms was acquired out of the space group *Imma* by decreasing the symmetry down to space group *Imm*2, followed by the removal of one atomic position of boron (2*b* (0,,*z*) *z* = 0.4708, Figure 3).

During the structure relaxation of NiPtB_2–*x*_, the crystallographic parameters were optimized and are presented in Table 4. The cell parameters obtained as the result of full relativistic as well as scalar relativistic calculations are in very good agreement with experimental results (differences of ∼1%).

**Table 4:** Atomic Coordinates and Cell Parameters of NiPtB_2–*x*_ Obtained as a Result of a Cell Relaxation Procedure (Space Group *Imm*2, no. 44)

	NiPtB_1.5_^a^	NiPtB_1.5_^b^	NiPtB_1.5_^c^
*a* (Å)	3.00656	3.01919	2.9835(3)
*b* (Å)	3.01434	2.99534	3.0470(3)
*c* (Å)	15.21831	15.2587	15.384(1)
Pt1 in 2*a* (0,0,*z*)	*z* = 0.07653	*z* = 0.07732	*z* = 0.07549
Pt2 in 2*b* (0,,*z*)	*z* = 0.92446	*z* = 0.92442	*z* = 0.92451
Ni1 in 2*a* (0,0,*z*)	*z* = 0.68388	*z* = 0.68466	*z* = 0.68390
Ni2 in 2*b* (0,,*z*)	*z* = 0.31496	*z* = 0.31401	*z* = 0.31610
B1 in 2*a* (0,0,*z*)	*z* = 0.21817	*z* = 0.21914	*z* = 0.21760
B2 in 2*b* (0,,*z*)	*z* = 0.78349	*z* = 0.78446	*z* = 0.78240
B3 in 2*a* (0,0,*z*)	*z* = 0.52815	*z* = 0.52559	*z* = 0.52920

a Calculated without spin–orbit coupling (SOC).

b With SOC.

c Experimental values, derived through transformation from space group *Imma* according to group-subgroup scheme shown in Figure 3.

**Figure 4:**
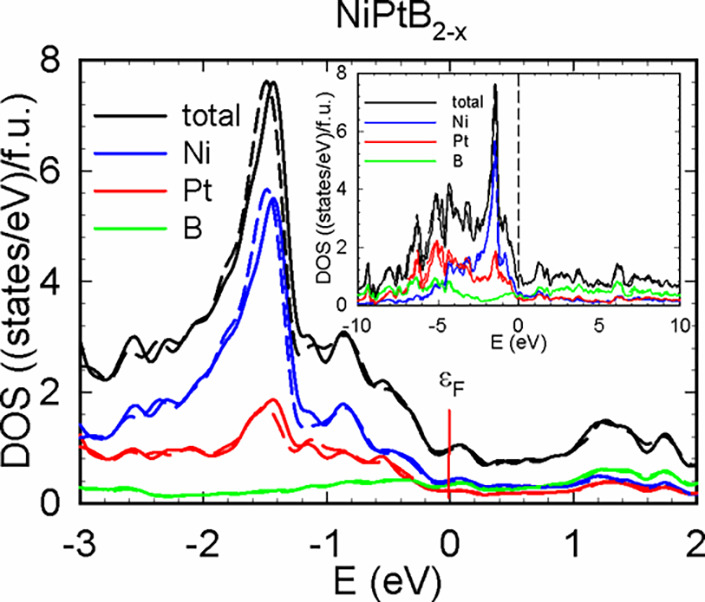
Electronic density of states of NiPtB_2–*x*_ in the vicinity of the Fermi level. Solid and dashed lines correspond to the values calculated without and with SOC, respectively. Inset represents the electronic density of states of NiPtB_2–*x*_ in a larger energy interval.

The electronic density of states of NiPtB_2–*x*_, calculated both with and without spin–orbit coupling, is presented in Figure 4. In both cases, in the valence band of NiPtB_2–*x*_ around −1.5 eV, the eDOS exhibits a sharp peak of around 8 states/eV f.u., constituted by *d*-states of nickel together with *d*-states of Pt. Around the Fermi level, the density of states is considerably lower, totaling around 1 states/eV f.u. For calculations performed with scalar relativistic and full relativistic pseudopotentials, the density of states at the Fermi level is dominated by states of Ni, with Pt and boron contributing 0.22 and 0.32 states/eV f.u., respectively, in both the scalar relativistic and full relativistic cases. Above the Fermi level, the density of states remains below 2 states/eV f.u. in the energy region from 0 to 10 eV (see inset) and is dominated by states of boron. In general, spin–orbit coupling has only a very modest impact on the electronic density of states in the vicinity of the Fermi level.

**Figure 5:**
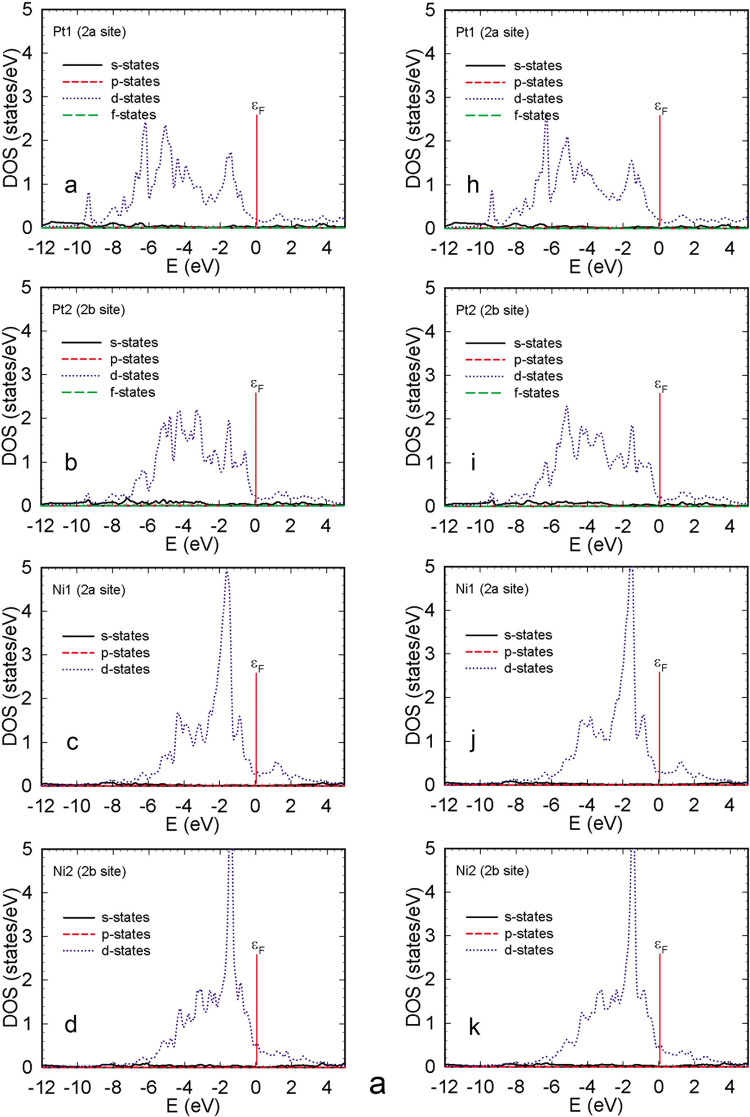

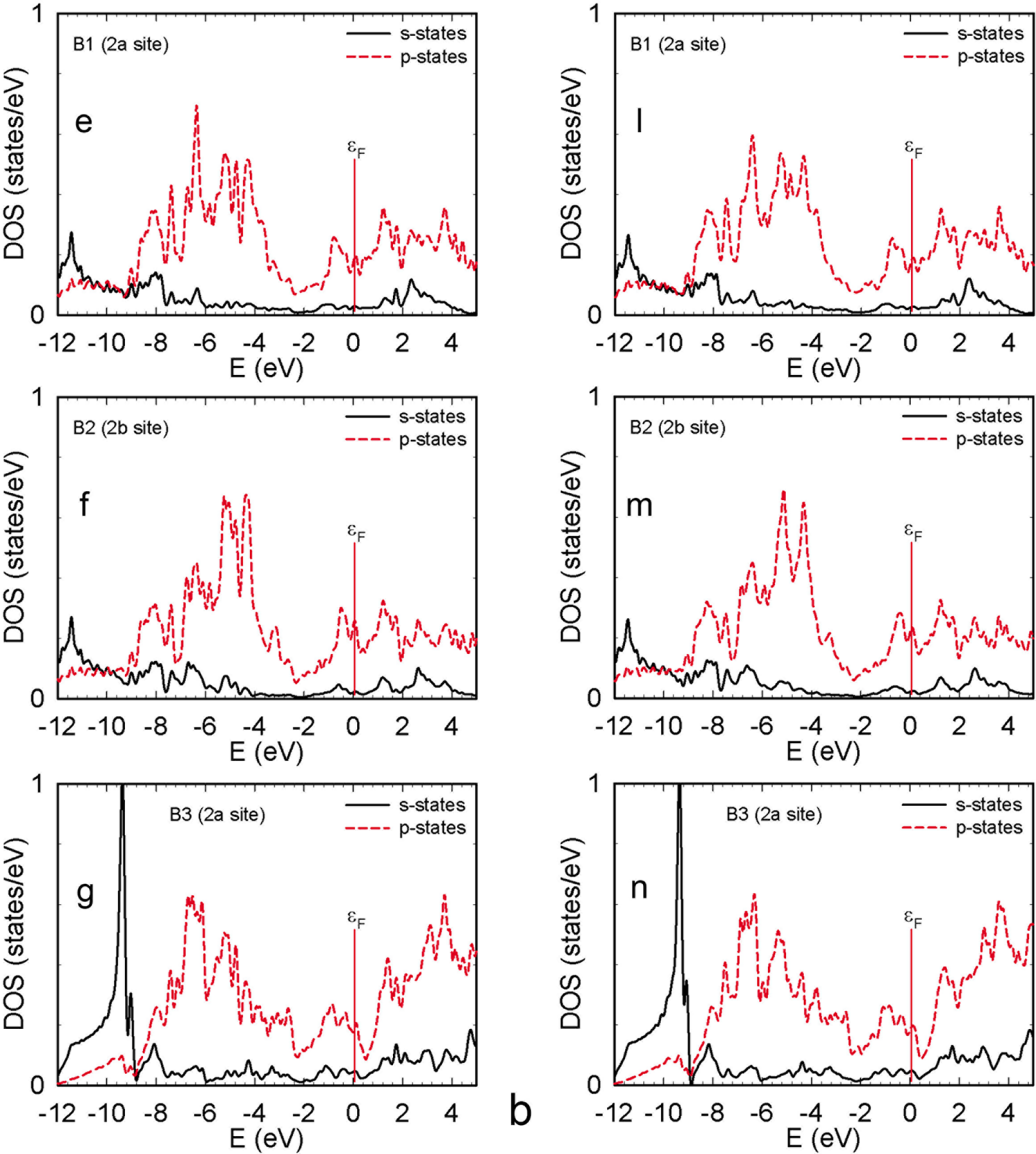
(a) Distribution of the partial (per one atom) density of states of Ni and Pt atoms in NiPtB_2–*x*_ without SOC (a–d) and with SOC (h−k). (b) Distribution of the partial (per one atom) density of states of boron atoms in NiPtB_2–*x*_ without SOC (e−g) and with SOC (l−n).

The distribution of the partial eDOS in NiPtB_2–*x*_ is presented in Figure 5a,b. In the valence band region, the eDOS of platinum and nickel atoms in both 2*a* and 2*b* Wyckoff positions is dominated by the respective *d*-states. Spin–orbit coupling does not influence the overall shape of the partial density of states of the above-mentioned atoms. The *d*-states of both platinum atoms (at the 2*a* and 2*b* positions) are spread more or less evenly in the valence band region, while nickel atoms are characterized by a distinct maximum of around 5 states /eV at −2 eV in their *d*-band.

The partial eDOS of states of B1 and B2 atoms is dominated by *p*-states both above and below the Fermi level. B3, on the other hand, exhibits a sharp peak of around 1 state/eV in its *s*-states, around −9 eV, both for calculations with and without spin–orbit coupling. This is attributed to a strong covalent bond with the Pt1 atom (corresponding to an equivalent peak in the density of *d*-states of Pt1). Such a response of the partial electron density of B3 closely resembles that found for boron atoms in binary Pt_2_B; however, in the case of the binary structure, Pt atoms do not display a corresponding anomaly in their partial eDOS (Figure S2). Detailed graphical data concerning the electronic density of states and numerical data on the relaxed crystal structure of Pt_2_B can be found in the Supporting Information (Figures S3, S4 and Table S1).

**Figure 6:**
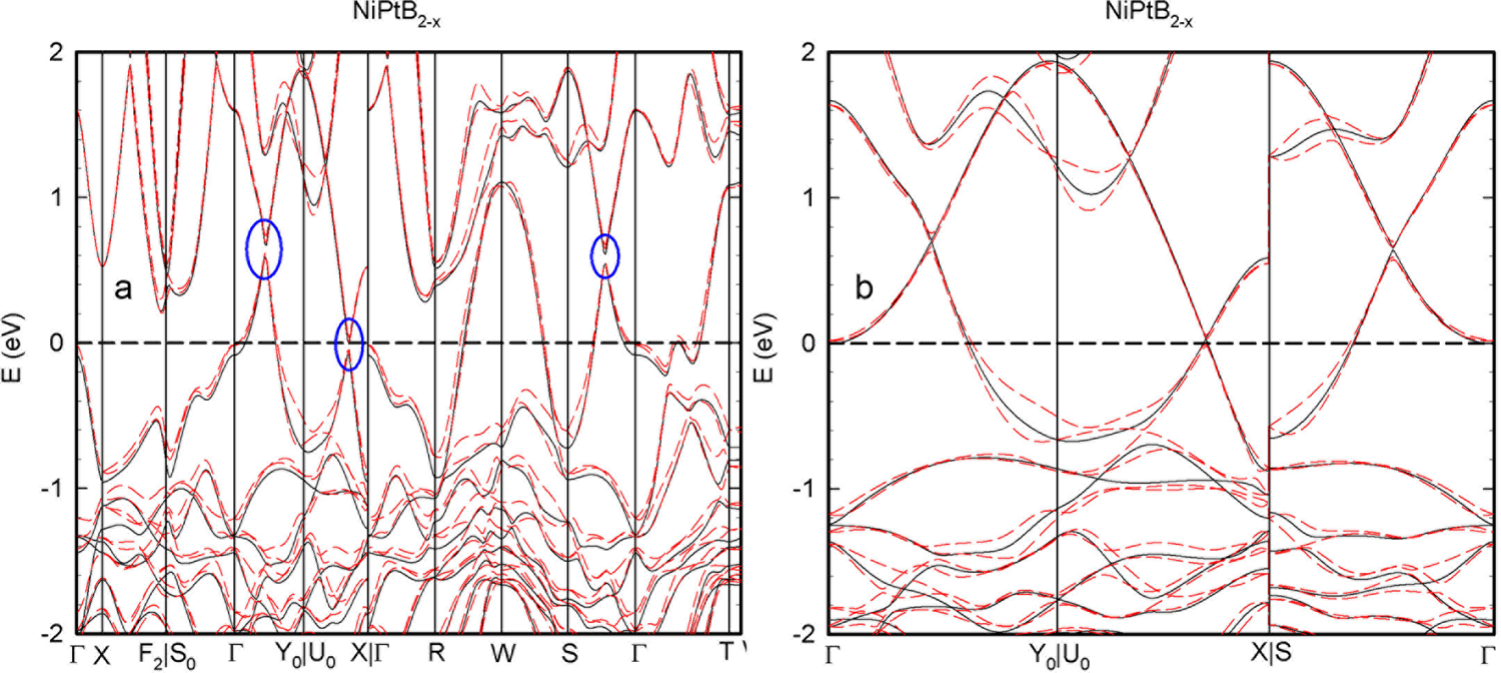
Electronic band structure of NiPtB_2–*x*_ calculated following the Γ—X—F_2_|S_0_—Γ—Y_0_|U_0_—X|Γ—R—W—S—Γ—T—W path. Blue ellipses point to the regions of interest described in text (a). The same band structure following the Γ—Y_0_|U_0_—X|S—Γ path was calculated with higher precision (b). On both graphs, solid and dashed lines correspond to the values calculated without and with SOC, respectively.

The electronic band structure for NiPtB_2–*x*_ was calculated following the Γ—X—F_2_|S_0_—Γ—Y_0_|U_0_—X|Γ—R—W—S—Γ—T—W path and is shown in Figure 6, where solid vertical lines correspond to the mentioned sequence of high-symmetry points. The electronic band structure of NiPtB_2–*x*_ is characterized by several bands crossing the Fermi level, hinting the material to be metallic, with three regions of interest marked with ellipses in Figure 6a. A calculation with higher precision was performed along the Γ—Y_0_|U_0_—X|S—Γ path and is presented in Figure 6b. As shown in Figure 6b, while the anomaly in Γ—Y_0_ is a band crossing point, the other two are of Dirac nature, demonstrating the linear dispersion of energy next to the avoided crossing. While in the U_0_—X region, spin–orbit coupling leads to a very modest splitting of bands to an amount of 0.035 eV; in the S—Γ region, the spin–orbit coupling induced anticrossing amounts to 0.07 eV.

### 3.3 Electron Localization Function

**Figure 7:**
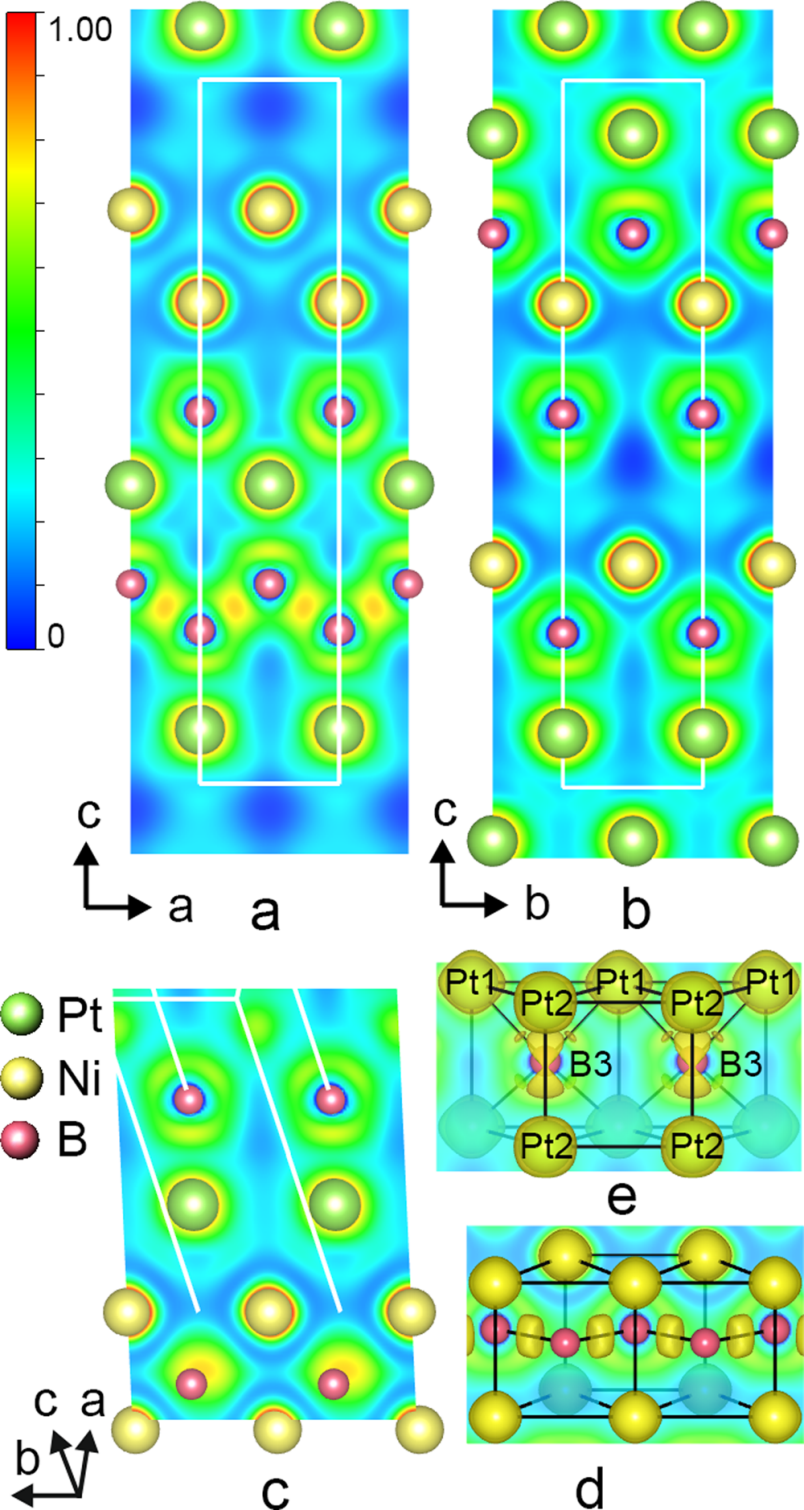
Sections of calculated ELF in NiPtB_2–*x*_ visualizing bonding in the lattice planes (010) (a), (100) (b), and between nickel and platinum layers (c). Distributions of the electron localization function in trigonal prisms: [BNi_6_] at ELF 0.710 (d) and [BPt_6_] at ELF 0.65 (e). Atom numbers correspond to those given in Table 4.

To visually illustrate the chemical bonding in NiPtB_2–*x*_, the distribution of the electron localization function (ELF) was calculated (Figure 7). As discussed above, the new boride structure is composed of three structural fragments, i.e., the nickel boride, platinum boride, and Pt–Ni substructures, which interconnect the boride substructures. The absence of electron accumulation between adjacent Ni and Pt atoms points to a mainly metallic character for the Ni–Pt bond. Directional covalent bonding in the zigzag −B–B– chains (ELF = 0.80) (Figure 7a,d) and polar Ni–B bonds (Figure 7b) can be clearly identified from the electron localization function distribution within the layer of [BNi_6_] trigonal prisms, similar to binary transition-metal monoborides.^52,53^ Bader charge analysis (Table 5) for atoms within the nickel boride substructure of NiPtB_2–*x*_ shows that Ni atoms donate electrons, which are involved in the formation of zigzag boron chains, and are therefore positively charged. ELF mapping in the platinum boride substructure revealed high localization domains between Pt and B (about 0.713 and 0.671) for shorter and longer Pt–B contacts (*d*_Pt-B_ = 2.20 Å; *d*_Pt-B_ = 2.25 Å) indicating covalent bonding in the Pt–B interactions (Figure 7e).

**Table 5:** Bader Charges of Atoms in NiPtB_2–*x*_ (Space Group *Imm*2) and Pt_2_B^a^

NiPtB_1.5_		Pt_2_B	
Pt1 in 2*a* (0,0,*z*)	–0.40	Pt1 in 4*i* (*x*,0,*z*)	–0.245
Pt2 in 2*b* (0,,*z*)	–0.41	Pt2 in 4*i* (*x*,0,*z*)	–0.189
Ni1 in 2*a* (0,0,*z*)	0.26	Pt3 in 4*i* (*x*,0,*z*)	–0.296
Ni2 in 2*b* (0,,*z*)	0.28	B1 in 4*i* (*x*,0,*z*)	0.486
B1 in 2*a* (0,0,*z*)	–0.04	B2 in 2*b* (0,,0)	0.487
B2 in 2*b* (0,,*z*)	–0.05		
B3 in 2*a* (0,0,*z*)	0.36		

a Bader charges are interpreted conventionally, as an additional charge on the atom.

**Figure 8:**
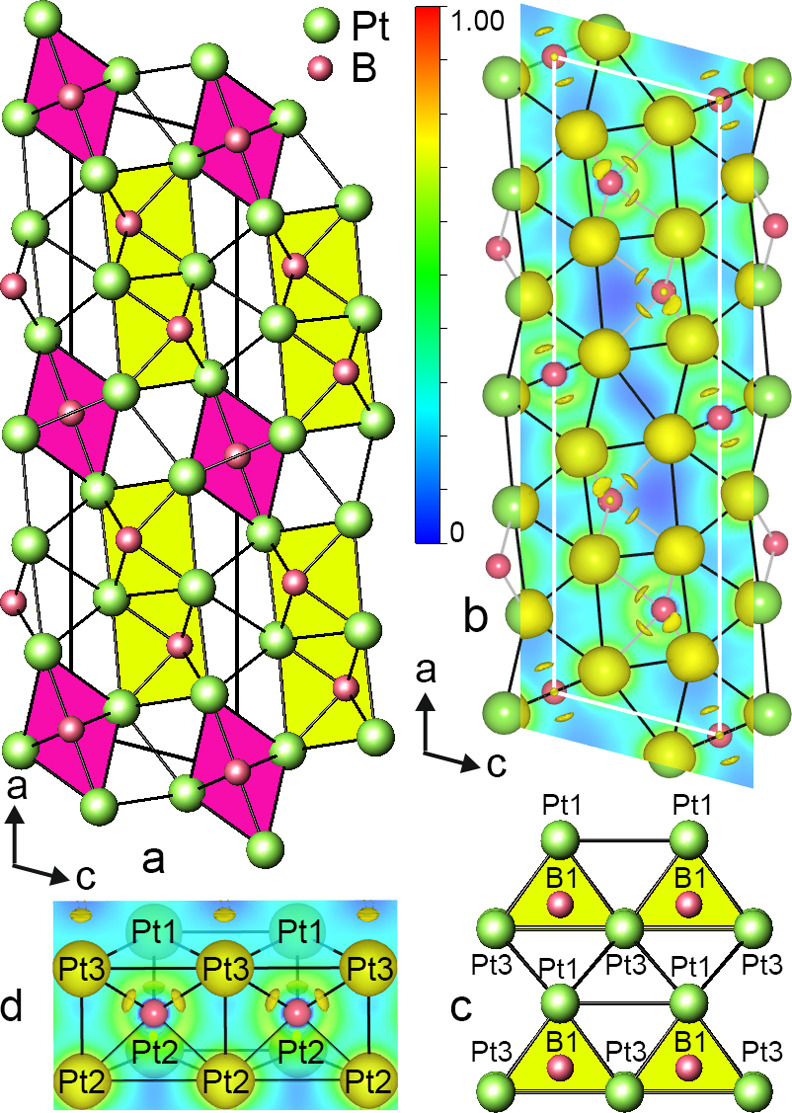
Crystal structure of Pt_2_B. Trigonal prisms [BPt_6_], green; octahedra [BPt_6_], red (a). Section of calculated ELF in Pt_2_B within the lattice plane (010) and isosurface at 0.65 (b). Layer of interlinked platinum trigonal prisms ( of a slab) in the structure of Pt_2_B (c). [B1Pt_6_] trigonal prism in the Pt_2_B: lattice plane through Pt2 and B1 atoms and isosurface at 0.65 (d). Atom numbers correspond to those given in Table S1.

As mentioned above in Section 3.1, the structures of NiPtB_2–*x*_ and Pt_2_B exhibit shared structural characteristics. Within Pt_2_B (Figure 8a,c), platinum trigonal prisms merge via square faces, forming continuous ribbons. These ribbons interconnect through triangular faces, producing slabs that extend infinitely along the *b*-axis. The slabs condense through Pt–Pt bonding, generating empty tetrahedral and octahedral voids, akin to CrB-type units. In the Pt_2_B structure, the layers originated from CrB-type alternate with layers of B-filled and empty platinum octahedra along the *a*-axis. Like the platinum boride subunits in NiPtB_2–*x*_, half of the [BPt_6_] trigonal prisms in Pt_2_B are vacant; however, the type of boron defect in the binary structure is ordered. Considering the ∼50% B occupation of Pt_6_ trigonal prisms, the platinum boride substructure in NiPtB_2–*x*_ qualitatively corresponds to the block of trigonal prisms in the Pt_2_B structure.

To evaluate the difference in chemical bonding of NiPtB_2–*x*_ and Pt_2_B, the distribution of the electron localization function was also calculated for the binary boride structure Pt_2_B. Although boron atoms have the same environment in both the trigonal prismatic [BPt_6_] slabs of the binary boride structure and the B-deficient platinum boride substructure of NiPtB_2–*x*_, the values of ELF between boron and platinum atoms in Pt_2_B are smaller (0.674–0.678) (Figure 8b,d). Similar distributions of ELF were found in [BPt_6_] octahedra in the binary platinum boride, indicating metallic bonding with a certain covalent contribution within these structural units. The analysis of Bader charges of atoms within the platinum boride substructure (both in the ternary and binary structures) supports the Pt–B bonding scheme characteristic for trigonal prismatic geometrical arrangements observed in the series of ternary platinum borides, e.g., YPt_x_B_6–2–*x*_^54^ and Sc_5_Pt_24_B_12_.^55^

### 3.4 Specific Heat

**Figure 9:**
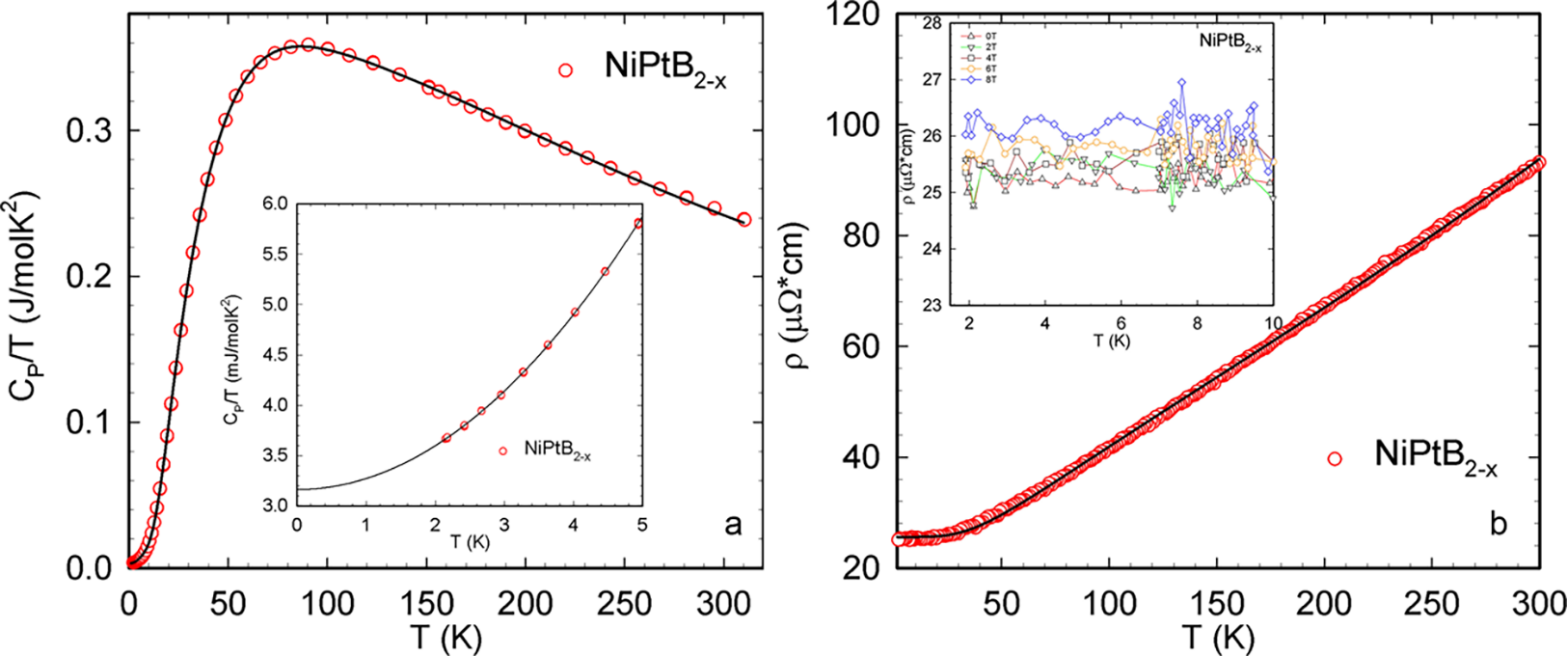
(a) Temperature-dependent specific heat, *C*_P_, of NiPtB_2–*x*_ in a form of *C*_P_(*T*)/*T*. Inset zooms on the low-temperature range of the graph below 5 K The solid line represents a least-squares fits according to text. (b) Temperature-dependent electrical resistivity, ρ, of NiPtB_2–*x*_. The solid line represents a least-squares fit according to the equation below. The inset shows the low-temperature electrical resistivity of NiPtB_2–*x*_ for different magnetic fields up to 8 T.

The specific heat *C*_P_(*T*) of NiPtB_2–*x*_ has been studied and is shown in Figure 9a (in the form of *C*_P_(*T*)/*T*). The lack of any anomalies in *C*_P_(*T*)/*T* for NiPtB_2–*x*_ indicates the absence of structural and electronic phase transitions in the measured temperature range. Within the low-temperature limit (*T* < 5 K), the heat capacity can be accounted for by a fit, yielding a Sommerfeld value γ = 3.16 mJ mol^–1^ K^–2^ and β = 0.109 mJ mol^–1^ K^–4^ (see inset Figure 9a). The former is slightly larger than the value of the electronic Sommerfeld coefficient, calculated from the electron density of states at the Fermi level, i.e., γ = 2.36 mJ mol^–1^ K^–2^. The β value corresponds to a low-temperature value of the Debye temperature θ_LT_ = 395 K.

In order to explore the vibrational properties of the lattice in an extended temperature range, the electronic contribution to the specific heat, *C*_el_ = γ*T*, was subtracted, and a model consisting of Debye and Einstein functions was adopted. The thermal expansion of the unit cell, especially at high temperatures, was not taken into account. Considering 3.5 atoms/f.u., the phonon dispersion relation of NiPtB_2–*x*_ consists of three acoustic and 7.5 optical branches. The former constitutes the Debye contribution, while the latter forms the Einstein part. As a result of the fit (solid line, Figure 9a), the Debye temperature θ_D_ = 247 K and three Einstein temperatures were obtained: *T*_E1_ = 110 K, *T*_E2_ = 336 K, and *T*_E3_ = 718 K with weights of *c*_1_ = 0.88, *c*_2_ = 2.42, and *c*_3_ = 4.2, respectively. To compare the Debye temperature derived from the fit with the one derived for low temperatures, the former has to be multiplied by *n*^1/3^ = 1.52, yielding 407 K (where *n* is the number of atoms per formula unit).

### 3.5 Electrical Resistivity and Hall Measurements

The temperature-dependent electrical resistivity of NiPtB_2–*x*_ has been studied using a 4-point method from room temperature down to 1.8 K. From the resistivity data, the compound behaves metallically without any transitions in the covered temperature range. The resistivity curve has been analyzed in terms of the Bloch–Grüneisen model,^56^ modified by a Mott–Jones term^57^ (−*AT*^3^) to account for the corrections due to scattering of conduction electrons on a narrow *d*-band (*s*-*d* scattering) in the vicinity of the Fermi energy,
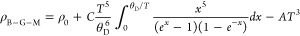
revealing a Debye temperature θ_D_ = 228 K, residual resistivity ρ_0_ = 25.6 μΩ cm, and a Mott–Jones coefficient *A* = 2.5 × 10^–7^ μΩ cm/K^3^. The compound is characterized by a low residual resistivity ratio (RRR) (RRR = ρ_300_/ρ_0_) value of 3.7, pointing to a rather high degree of disorder in the sample (see Figure 9b).

The magnetic field response of the electrical resistivity of NiPtB_2–*x*_ was studied in fields up to 8 T. The results are presented in the inset of Figure 9b. The electrical resistivity of NiPtB_2–*x*_ exhibits no significant field dependence, as expected for a compound lacking any magnetic correlations.

Hall measurement have been performed on the above-mentioned sample at *T* = 20 K*,* leading to a Hall coefficient value of . The sign of the Hall coefficient indicates that holes are the major charge carriers in NiPtB_2–*x*_.

## 4 Summary

A new ternary boride, NiPtB_2–*x*_ (*x* = 0.5), has been synthesized, and its crystal structure was determined from single-crystal and powder X-ray diffraction data. NiPtB_2–*x*_ is the first representative of borides crystallizing in a ternary derivative structure of the MoB type. This crystal structure can be described as the alternative stacking (along the [001] direction) of planar layers, composed of nickel boride and platinum boride trigonal prisms. The prism axes in subsequent layers are perpendicular to each other. Boron atoms within the nickel boride layer interlink to form infinite zigzag chains and together with nickel atoms produce CrB-type structural fragments. The platinum boride substructure exhibits disordered boron vacancies. Considering B-deficiency, the platinum boride substructure in NiPtB_2–*x*_ quantitatively corresponds to trigonal prismatic slabs in Pt_2_B which, however, displays ordered boron defects. Beside the structural relationships with the MoB structure, NiPtB_2–*x*_ exhibits common structural fragments with the Ru_2_ZnB_2–*x*_-type MAB phase and with the CuIr_2_B_2–*x*_-type. The configuration of the partial eDOS of Pt and B in the platinum boride substructure in NiPtB_2–*x*_ points to a strong covalent bond between these atoms. Bader charge analysis indicated that the formation of zigzag boron chains requires the nickel atoms to provide electrons, stabilizing the zigzag boron chains. Boron charge gains vary from positive to negative values with the crystallographic position, depending on metal atoms located in the neighborhood. Chemical bonding analysis by ELF distribution indicated covalent bonding within the boron zigzag chains and metallic bonding between neighboring Ni and Pt, as well as covalent bonding between Pt and B within trigonal prisms, both in NiPtB_2–*x*_ and Pt_2_B. Electrical resistivity and specific heat measurements of NiPtB_2–*x*_ indicate that the studied compound is a metal with a Sommerfeld value of γ = 3.16 mJ mol^–1^ K^–2^, typical for metals. A moderately high value of the residual resistivity ρ_0_ = 25.6 μΩ cm points to disorder in the sample that supposedly originated from the partial occupation of boron atomic sites. At *T* = 20 K, the compound is characterized by a positive Hall coefficient , suggesting that holes are the dominant charge carriers in the compound. The electronic density of states of NiPtB_2–*x*_ at the Fermi level is approximately 1 states/eV f.u.

Notably, NiPtB_2–*x*_ is located on the line in the Ni–Pt–B phase diagram extending from binary NiB to binary Pt_2_B. In the Ni–B binary system, NiB crystallizes in orthorhombic symmetry with the CrB prototype structure. Substitution of Pt/Ni is able to produce a new ternary structure, which combines structural fragments common to both early (CrB-type) and late transition metals (e.g., Pt_2_B). NiB is predicted to be a promising interphase material for future ultrahigh-temperature ceramic matrix composites.^58^ The electronic partition (polar covalent metal-boron bonds and strong B–B covalent bonds) observed from electronic structure calculations may presumably be responsible for the high bulk and shear modulus, in a similar manner to what has been shown in structurally related borides, e.g., CrB, NiB, and so forth. Due to metallic Ni–Pt bonding between boride subunits in NiPtB_2–*x*_, the compound may possess a unique combination of metal and ceramic properties. The results obtained in this work call for further studies aimed at hardness and elastic properties as well as catalytic properties of this new compound. Research on Ni(Pt,Ir)-A-B (A is *p*-element) ternary and quaternary compositions related to MAB phases is in progress.

## Supporting Information Available

Crystal structures of of Ru_2_ZnB_2–*x*_, CuIr_2_B_2–*x*_ and Ir_4_ZnB_3_; distribution of the partial (per one atom) density of states in Pt_2_B without and with SOC; atomic coordinates and cell parameters of Pt_2_B (space group *C*2/*m*) obtained as a result of cell relaxation procedure; electronic density of states of Pt_2_B at the vicinity of the Fermi level without and with SOC; electronic band structure of Pt_2_B (PDF)

## Notes

No uncommon hazards are noted derived from the experimental work carried out
