# Supplementary material for: New Layered Boride NiPtB2–x (x = 0.5) with a Ternary Derivative Structure of MoB
Source: Inorg Chem. 2025 Jan 27;64(5):2282–93. doi: 10.1021/acs.inorgchem.4c04399 (PMC11815825; doi:10.1021/acs.inorgchem.4c04399)
Supplement: Supplementary file 1 — ic4c04399_si_001.pdf [file ic4c04399_si_001.pdf]

SUPPORTING INFORMATION

New layered boride $NiPtB_{2-x}$ (x=0.5) with a ternary derivative structure of MoB

Leonid Salamakha[1,4], Oksana Sologub[1], Berthold Stöger[2], Herwig Michor[1], Neven Barisic[1,5], Peter F. Rogl[3], Ernst Bauer[1]

[1]Institute of Solid State Physics, TU Wien, A-1040 Vienna, Austria

[2]X-Ray Centre, TU Wien, A-1060 Vienna, Austria

[3]Institute of Materials Chemistry, University of Vienna, A-1090 Vienna, Austria

[4]Department of Physics of Metals, Faculty of Physics, I. Franko L'viv National University, 79005 L'viv, Ukraine

[5]Department of Physics, Faculty of Science, University of Zagreb, HR-10000 Zagreb, Croatia

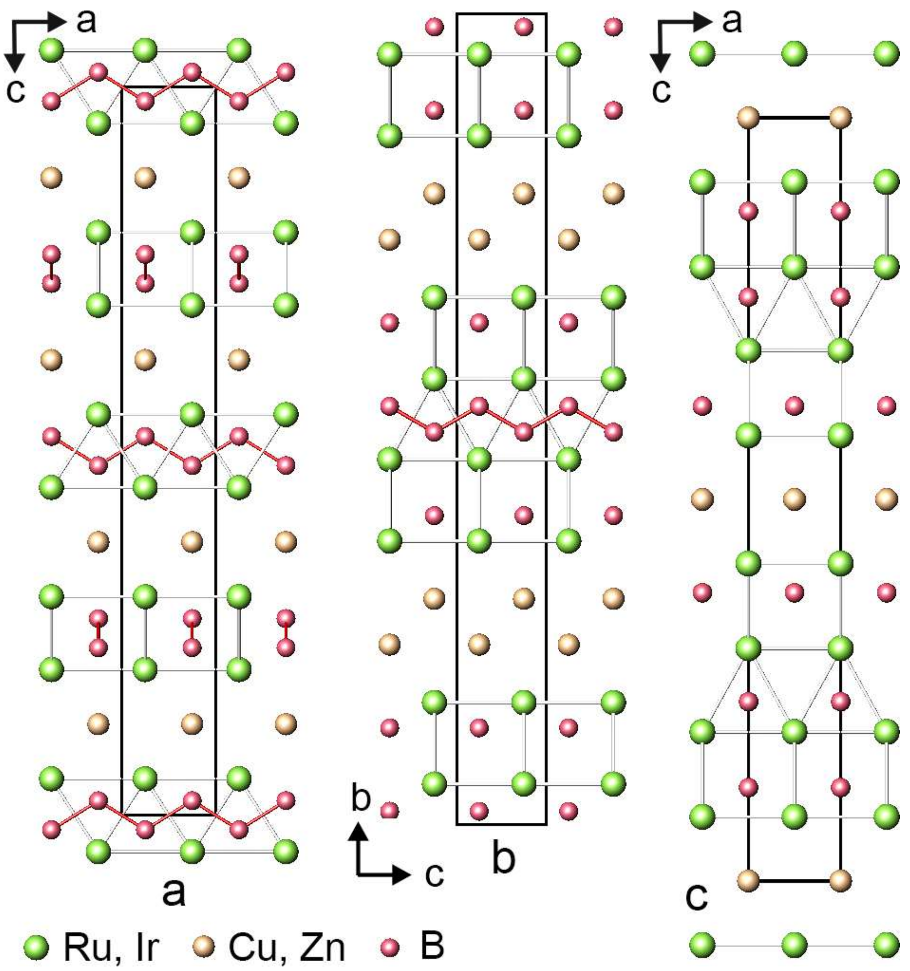

Figure S1. Crystal structures of Ru_2ZnB_{2-x}[a] (tentative visualization) (a), $CuIr_2B_{2-x}$[b,c] (b) and Ir_4ZnB_3[d] (c).

[a]W. Jung, K. Petry, Ternäre Boride des Rutheniums mit Aluminium und Zink. *Z. Kristallogr.* **1988**, *182*, 153-154.

[b]W. Klünter, W. Jung, The copper iridium boride $Cu_2Ir_4B_3$ with a layer structure derived from the $ZnIr_4B_3$ Type. *Z. Anorg. Allg. Chem.* **2000**, *626*, 502-505.

[c]O. Sologub, L.P. Salamakha, H. Michor, N. Barisic, S. Mudry, P.F. Rogl, E. Bauer, Cu-Ir-B system: Phase equilibria, crystal structure, bonding and electronic structure of compounds. J. Solid State Chem. **2025**, *344*, 125176.

[d]K. Petry, W. Jung, Darstellung und Kristallstrukturen der Zink-Iridiumboride $ZnIr_4B_3$, $Zn_6Ir_{11}B_{6-x}$, $ZnIr_2B_2$ und Zn_2Ir_2B. *J. Alloys Compd.* **1992**, *183*, 363-376.

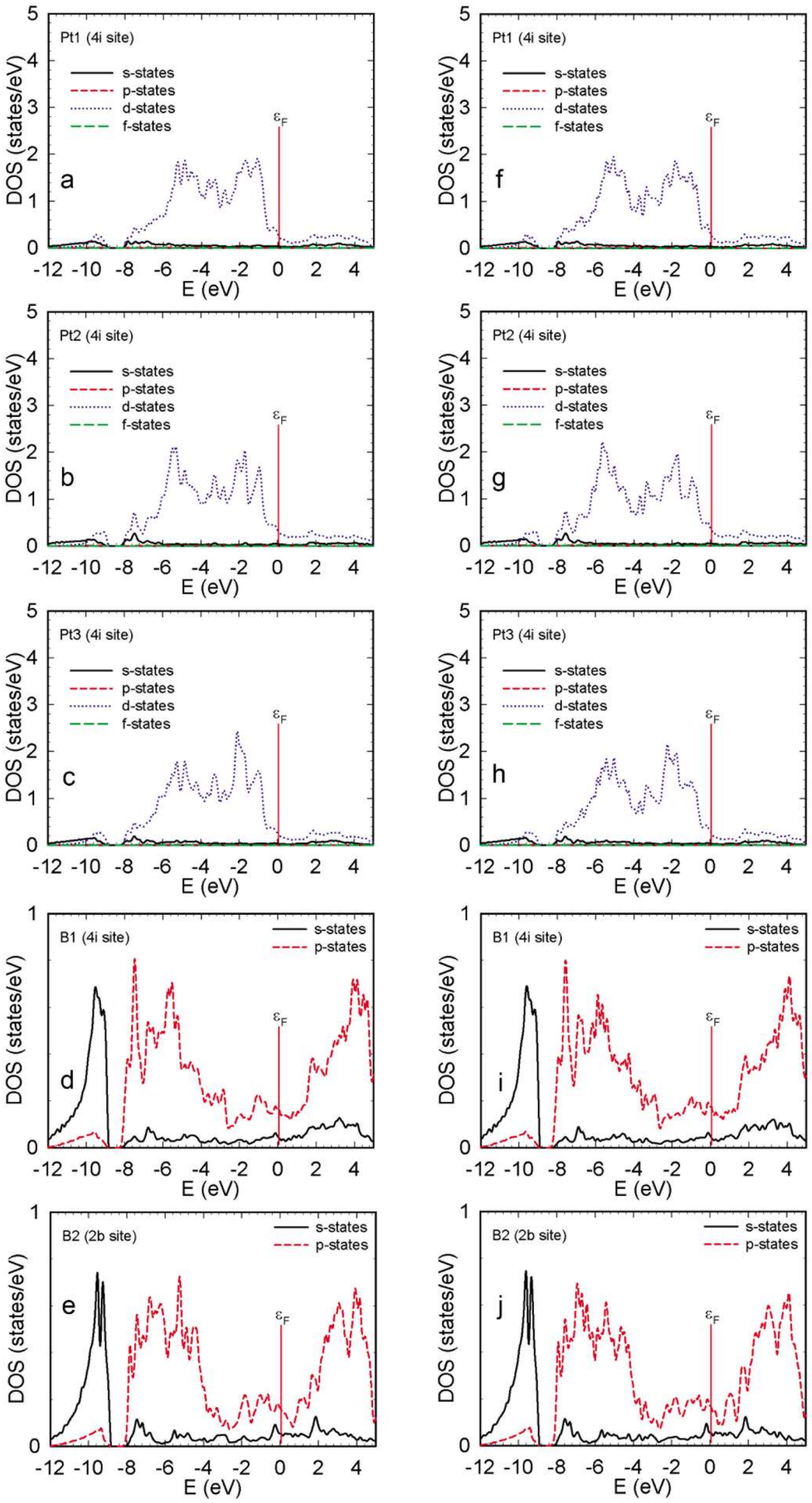

Figure S2. Distribution of the partial (per one atom) density of states in Pt_2B without (a, b, c, d, e) and with (f, g, h, i, j) SOC.

Table S1. Atomic coordinates and cell parameters of Pt_2B (space group *C*2/*m*) obtained as a result of cell relaxation procedure.

	Pt_2B without SOC	Pt_2B with SOC	Pt_2B experimental[a]
a, Å	16.18854	16.18670	16.2717(11)
b, Å	3.29552	3.30185	3.2788(2)
c, Å	4.36018	4.35017	4.4200(3)
β, °	104.6798	104.7050	104.401(4)
Pt1 in 4*i* (*x*,0,*z*)	*x*=0.05380	*x*=0.05392	*x*=0.05365(4)
	y=0.30249	*y*=0.30293	*z*=0.3011(1)
Pt2 in 4*i* (*x*,0,*z*)	*x*=0.22571	*x*=0.22546	*x*=0.22523(4)
	y=0.24423	*y*=0.24379	*z*=0.2443(1)
Pt3 in 4*i* (*x*,0,*z*)	*x*=0.38875	*x*=0.38880	*x*=0.38874(4)
	y=0.16548	*y*=0.16551	*z*=0.1682(1)
B1 in 4*i* (*x*,0,*z*)	*x*=0.17450	*x*=0.17514	*x*=0.177(1)
	y=0.66222	*y*=0.66333	*z*=0.653(4)
B2 in 2*b* (0,½,0)	0,½,0	0,½,0	0,½,0

[a] O. Sologub, L. Salamakha, P. Rogl, B. Stöger, E. Bauer, J. Bernardi, G. Giester, M. Waas, Svagera, Pt–B system revisited: Pt_2B, a new structure type of binary borides. Ternary WAl_{12}-type derivative borides. *Inorg. Chem.* **2015**, *54*(22) 10958–10965.

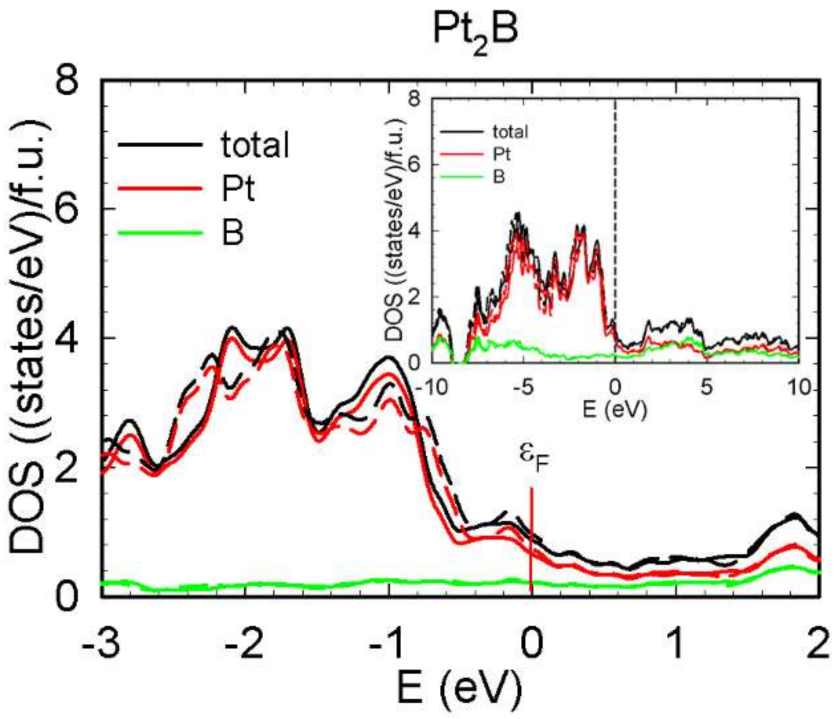

Figure S3. Electronic density of states of Pt_2B at the vicinity of the Fermi level. Solid and dashed lines correspond to the values calculated without and with SOC respectively. Inset represents the electronic density of states of Pt_2B in a larger energy interval.

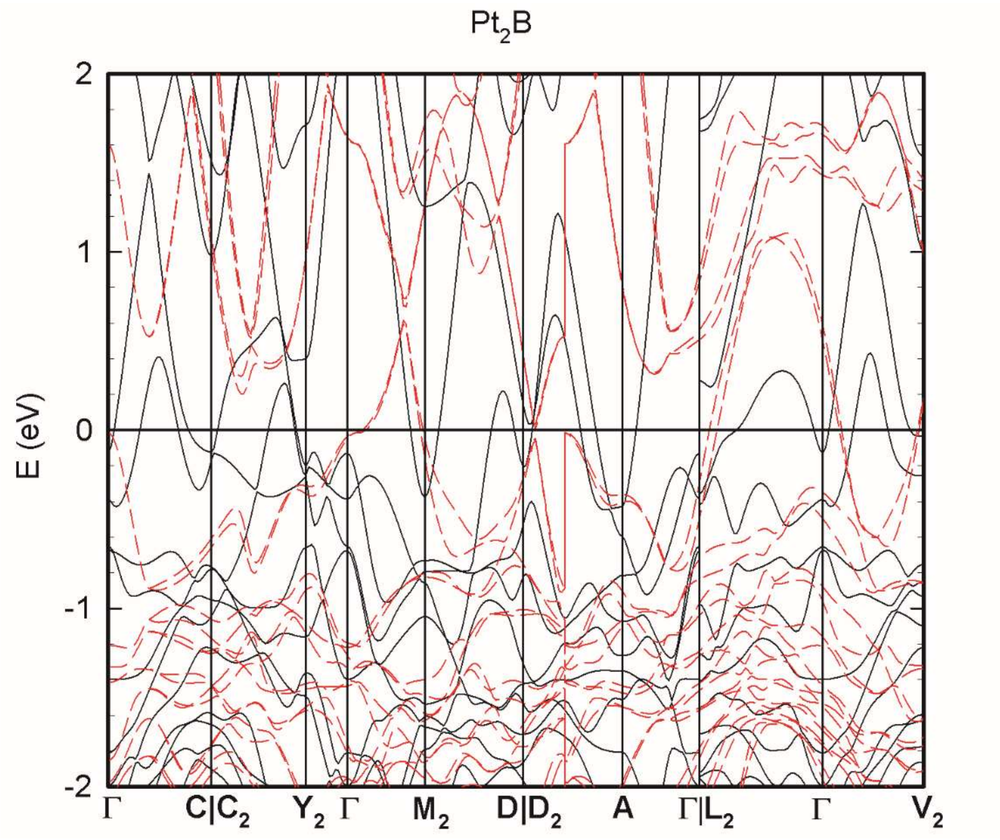

Figure S4. Electronic band structure of Pt_2B. Solid and dashed lines correspond to the values calculated without and with SOC respectively.
